# Role of protein kinases CK1α and CK2 in multiple myeloma: regulation of pivotal survival and stress-managing pathways

**DOI:** 10.1186/s13045-017-0529-5

**Published:** 2017-10-02

**Authors:** Sabrina Manni, Marilena Carrino, Francesco Piazza

**Affiliations:** 1 Department of Medicine, Hematology Section, University of Padova, Via Giustiniani 2, 35128 Padova, Italy; 2 Venetian Institute of Molecular Medicine, Padova, Italy

**Keywords:** Multiple myeloma, CK1, CK2, Bone marrow microenvironment, Survival signaling pathways, Non-oncogene addiction

## Abstract

Multiple myeloma (MM) is a malignant tumor of transformed plasma cells. MM pathogenesis is a multistep process. This cancer can occur de novo (rarely) or it can develop from monoclonal gammopathy of undetermined significance (most of the cases). MM can be asymptomatic (smoldering myeloma) or clinically active. Malignant plasma cells exploit intrinsic and extrinsic bone marrow microenvironment-derived growth signals. Upregulation of stress-coping pathways is also instrumental to maintain MM cell growth. The phylogenetically related Ser/Thr kinases CSNK1A1 (CK1α) and CSNK2 (CK2) have recently gained a growing importance in hematologic malignancies arising both from precursors and from mature blood cells. In multiple myeloma, CK1α or CK2 sustain oncogenic cascades, such as the PI3K/AKT, JAK/STAT, and NF-κB, as well as propel stress-related signaling that help in coping with different *noxae*. Data also suggest that these kinases modulate the delivery of growth factors and cytokines from the bone marrow stroma. The “non-oncogene addiction” phenotype generated by the increased activity of CK1α and CK2 in multiple myeloma contributes to malignant plasma cell proliferation and survival and represents an Achilles’ heel for the activity of small ATP competitive CK1α or CK2 inhibitors.

## Background

Multiple myeloma (MM) is the second most frequent hematologic malignancy, accounting for about 13% of all blood cancers [1]. The bulk of the tumor is represented by transformed plasma cells (PCs), which accumulate firstly in the bone marrow (BM) in a microenvironmental *niche* that supports their growth [2]. Along with the disease progression, malignant PCs lose their dependency from the BM and gain features of autonomous growth with widespread dissemination of plasma cell leukemia [3, 4]. Clinically, MM is characterized by target organ damage, with anemia, renal insufficiency and bone resorption/loss with bone pain, hypercalcemia, and pathological fractures. In addition, MM patients develop immune-paralysis with hypogammaglobulinemia and susceptibility to infections [5].

The molecular and genetic features of MM have lately been described. MM are divided in cases bearing chromosomal translocations affecting the IgH locus (30%); cases with hyperdiploidy (trisomies) of odd chromosomes 3, 5, 7, 9, 11, 15, 17, and 19 (40–45%); cases with both the alterations (15%); and cases with other abnormalities (10–15%) [6, 7]. The genomic analysis of MM cases has revealed a complex genetic architecture that suggests a continuous clonal evolution in a Darwinian process and few recurrent mutations concentrated in clusters of genes, which regulate, among others, the translation process, chromatin modification and gene transcription, including the nuclear factor kappa-light-chain-enhancer of activated B cells (NF-κB) pathway [6].

Besides intrinsic alterations in MM PCs, an aberrant BM microenvironment participates in MM pathogenesis. The stromal *niche* surrounding malignant PCs is able to deliver trophic signals represented by cytokines, such as interleukin-6 (IL-6) and tumor necrosis factor-α (TNF-α), growth factors, such as insulin-like growth factor-I (IGF-I) and related proteins, and soluble glycoproteins, such as Wnt and Hedgehog. All these signaling cascades inside MM cells shift the *milieu* towards osteoclast propelling features and promote aberrant neoangiogenesis [2, 8]. Therefore, MM cells and bone marrow stromal cells (BMSCs) depend on a number of signaling cascades whose regulation is still largely unknown. MM cells rely also on intracellular pathways that have the ability to manage a different array of stresses, including the proteotoxic, replicative, and oxidative stress [9–11]. Thus, molecules acting as “stress managers” may become essential for the optimal fitness of malignant PCs. Examples are the transcription factor IRF4, which is part of a rewired transcriptional program in malignant PCs as compared to normal counterparts [12], the kinase ATR [13], and the scavenger enzyme SOD2 [14].

Mutations as well as hyper-function of certain fundamental proteins and cascades may cause tumor promotion and progression. In this context, it is not doubtable that native or newly generated protein kinases (PK) may become pivotal players. As a proof of concept that PK may be central in oncogenesis is the clear evidence of the lethal consequences for many tumor types caused by their inhibition. Some examples are the drugs imatinib, gefitinib, ibrutinib, or fostamatinib, which target a number of receptor/cytosolic tyrosine kinases and have proven to be clinically effective therapeutic options for solid tumors, chronic myeloid leukemia (CML) or chronic lymphocytic leukemia (CLL), and non-Hodgkin lymphomas. However, despite the fact that in several B cell malignancies protein kinases represent valid therapeutic targets, this proof of principle is lacking in MM.

In this regard, among protein kinases driving MM cell survival, recently, the Ser/Thr kinases CK1α and CK2 have been shown to play an important role as regulators of signal transduction and stress response [15–17]. We will herein review CK1α and CK2 function in MM and discuss the potential of targeting their kinase activity as a suitable therapeutic strategy for this B cell-derived tumor.

### Protein kinase CK1α: emerging roles in cancer

CK1α belongs to a family of highly conserved monomeric Ser/Thr kinases composed by seven members encoded by different genes (α, β, γ1, γ2, γ3, δ, and ε), displaying the highest homology in their kinase domains (50–90% identical) with similar substrate specificity. CK1 members regulate membrane biology, molecular transport, signal transduction, transcription, translation, and DNA damage response [18, 19]. In the last few years, CK1α, encoded by the *CSNK1A1* gene, has been involved in cancer with a role that seems multifaceted.

CK1α inhibition, leading to stabilization of β-catenin, acts as a tumor promoter in the absence of p53 in intestinal epithelial cells, while its inactivation does not turn into tumor formation as long as p53 is active [20, 21]. Nevertheless, CK1α is a tumor promoter in acute myeloid leukemia (AML), provided there is an intact p53 [22]. CK1α has been shown to negatively regulate Ras-induced autophagy in models of Ras-driven transformation by controlling the phosphorylation of FOXO3A on S318/321 and its subsequent nuclear extrusion [23].

Other reports have involved CK1α in tumors. CK1α is a tumor supporter in diffuse large B cell lymphoma (DLBCL) of activated B cell subtype, inducing the activation of NF-κB through the regulation of the CBM1 complex (CARD11, BCL10, MALT1) [24]. Mutations of CK1α have been detected in melanoma, clear cell renal cell carcinoma [25, 26], colon cancer [27], esophageal adenocarcinoma [28], adult T cell leukemia/lymphoma [29], and del(5q) myelodisplatic syndromes (MDS) [30]. Schneider and colleagues demonstrated a prominent role of CK1α in del(5q) MDS. Our group and others have demonstrated an oncogenic role of CK1α in MM [17, 31].

Altogether, a growing body of data points to a potential role for CK1α in carcinogenesis in different tumor types.

### Protein kinase CK2: an indispensable molecule for cancer cell survival

Protein kinase CK2 is a Ser/Thr kinase that regulates critical cellular processes. It is composed by a tetramer of two catalytic (α or α’) and two regulatory subunits (β) [32]. The substrate specificity is believed to rely on the β subunit even though tetramer-independent functions of the α and β moieties have been recognized. CK2 has been involved in cell proliferation, apoptosis, transcription and translation, adhesion and motility, and stress-coping [33–35]. CK2 has hundreds of substrates [36], among which are pivotal oncogenic proteins, like AKT [37, 38], c-Myc [39], NF-κB [8, 40], and signal transducer and activator of transcription 3 (STAT3) [16]. Given its multilateral involvement in cell biology, it is not surprising that high CK2 activity and expression have been correlated to poor prognosis and resistance to anti-cancer agents in different types of cancers, including breast [41], lung [42], prostate [43], renal [44], bladder carcinoma [45], melanoma [46], and hematological malignancies [47]. CK2 acts as a typical “non-oncogene addiction” molecule, since cancer cells strongly rely on it even though it is generally not altered by typical mutations leading to a gain of function phenotype [47, 48]. However, recently, CK2β deletions were identified in 15% of cases of early relapsing DLBCL patients compared to those of late relapsing [49].

The mechanisms by which CK2 sustains malignant tumor growth are numerous. CK2 promotes cancer cell survival by activating tumor-promoting oncogenes (such as c-Myc [39] in lymphoma, Ras-ERK [50] in melanoma [46], AKT [51–54] in bladder, colon, and blood cancers) or by inhibiting tumor suppressors (such as PTEN) [53, 55].

CK2 also strongly supports the activity of important signaling cascades, such as the NF-κB, in breast cancer [56] as well as in multiple myeloma, lymphoma [16] and leukemia [54], Wnt-β-catenin, [57], Hedgehog in pleural mesothelioma [58], and STAT3-dependent signaling in solid and in hematological tumors [34]. CK2 phosphorylates NF-κB RelA/p65 on Ser529, increasing p65 transcriptional activity downstream external stimuli [59]. CK2 positively modulates STATs by phosphorylating Ser727 of STAT3 [16]. This kinase regulates β-catenin stability [60, 61] and Gli1 function [62]. Besides its role as a signaling regulator, CK2 is involved in cellular processes of stress-coping/fitness augmentation. For instance, CK2 regulates the DNA damage response [63, 64], autophagy [65], and endoplasmic reticulum (ER) stress [15, 66]. A major role in cell survival is believed to rely on CK2 modulation of caspase activity [67–69].

### CK1α and CK2 in multiple myeloma: regulation of signal transduction

Others and our group demonstrated that CK1α is a pro-growth kinase in MM. In the work by Hu et al. [31], CK1α was found to promote survival and proliferation of MM cell lines and cMyc/KRasV12-transduced BaF3 cells in xenograft mouse models. CK1α inhibition led to higher interferon-α and TNF-α signaling. More recently, we demonstrated that CK1α is highly expressed in the vast majority of MM patient PCs (in a large microarray data set series) compared to that in healthy PCs [17]. CK1α loss of function with a pan-CK1 inhibitor (D4476) or RNA interference (RNAi) was accompanied to MM cell apoptosis, cell cycle arrest and downregulation of β-catenin, and AKT survival signaling, in a mechanism that could involve caspase and p53 [17]. Of note, CK1α inactivation was able to overcome BMSCs protection. Moreover, CK1α inhibition synergically boosted bortezomib and lenalidomide cytotoxicity. Interestingly, lenalidomide treatment of MM cells determined a deregulation of CK1α expression, in a time- and dose-dependent manner, in a mechanism similar to that observed in other cell types [70].

CK2 is overexpressed and enzymatically more active in malignant MM PCs from patients and cell lines compared to that in healthy controls [71]. CK2 was found localized in the cytoplasm, in the nucleus, and in a small fraction also in the ER of malignant PCs [15]. The use of ATP competitive CK2-specific inhibitors like tBB, K27, and the newly developed clinically graded CX-4945 (silmitasertib) caused malignant PCs apoptosis, being less toxic to the non-malignant counterparts [8, 15, 16, 71]. From a molecular standpoint, CK2 inactivation with chemicals or by RNAi in MM impacted on two main signaling pathways, the NF-κB and the JAK-STAT cascades, which are known to exert fundamental roles in MM pathogenesis. CK2 blockade was associated to an accumulation of the inhibitor of NF-κB (IκBα) at baseline conditions as well as upon a strong NF-κB-activating stimulus, such as TNF-α. Consequently, the transcriptional activity of NF-κB was found substantially compromised. Moreover, CK2 inhibition led to an impaired phosphorylation of STAT3 on Tyr705 and Ser727. Growth stimuli, such as IGF-I and IL-6, were not able to overcome the lower cell survival frequency consequent to CK2 inhibition, suggesting a central role for this protein kinase downstream manifold signaling pathways. As a result, myeloma cells with less active CK2 were much more sensitive to the cytotoxic effect of a chemotherapeutic drug employed in MM, such as melphalan or the new-generation drug proteasome inhibitor bortezomib.

**Fig. 1:**
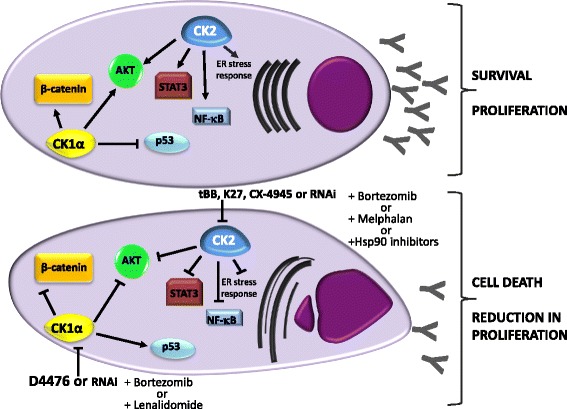
CK1α or CK2 targeting determines MM plasma cell apoptosis through the deregulation of important MM survival signaling pathways. CK1α promotes β-catenin and AKT signaling while negatively control p53 in MM. Its inhibition (with D4476 or by RNAi) alone or in association with bortezomib or lenalidomide impinges on the activation of these survival events, promoting MM plasma cell death. CK2 sustains plasma cell survival and proliferation through the regulation of NF-κB, STAT3, and AKT. Its inactivation (with chemical compounds such as tBB, K27, CX-4945, or RNAi) alone or in association with old (melphalan) or new agents (bortezomib, Hsp90 inhibitors) causes plasma cell apoptosis and reduction in proliferation

The role of CK1α and CK2 on MM survival signaling events and on response to drugs is summarized in Fig. 1.

### CK1α and CK2 in multiple myeloma: regulation of homeostatic/stress response pathways

**Fig. 2:**
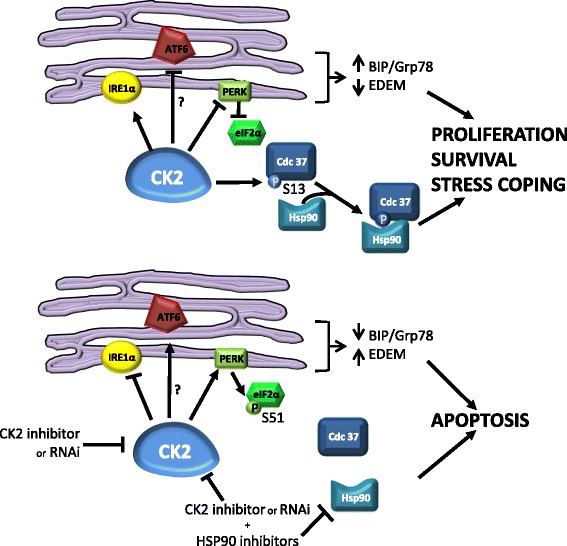
Schematic representation of the role of CK2 on ER stress response. CK2 promotes a compensatory UPR by stabilizing IRE1α, inhibiting the kinase PERK and, consequently, the phosphorylation of Ser51 (S51) on EIF2α. Moreover, CK2, by phosphorylating Ser13 (S13) of Cdc37, tightens its association with chaperones, leading to MM plasma cell survival, proliferation, and enhanced stress-coping ability. CK2 inactivation (chemically or with gene silencing) leads to a deregulation of the above cascades, inducing MM cell apoptosis

A mechanism accounting for CK2-driven regulation of multiple signaling cascades is the one described by Miyata [72, 73]. CK2 phosphorylates the co-chaperone Cdc37 on Ser13. This phosphorylation enables Cdc37 to tighten its association with the chaperone Hsp90 and with a number of client protein kinases, many of which are important in the signal transduction across different proliferative and survival pathways. Thus, CK2 exerts a “mastermind-like” control on many cellular functions. This role is believed to be exquisitely important in the context of malignant transformation, where signaling modules are overexploited by an increased proliferation/survival/stress-coping need. In particular, in MM, the ER stress induced unfolded protein response (UPR) aimed at coping with unfolded protein load in the ER. UPR can end up in a compensatory response or (if the ER stress is prolonged/overwhelming) in apoptosis. Analyzing whether CK2 could impact on the proteotoxic/unfolded protein stress in MM, we found that a fraction of CK2 is localized in the ER and when ER stress was elicited by thapsigargin, the CK2 kinase activity raised [15]. Upon CK2 inactivation, we observed a number of changes in the homeostatic molecules regulating the ER stress/UPR: a decreased expression of the co-chaperone Bip/Grp78 and of the kinase/endoribonuclease IRE1α (which was also less phosphorylated), but an activation of the kinase PERK (which was instead more phosphorylated at Thr981) and of the downstream eukaryotic initiation factor 2-eIF2α (more phosphorylated on Ser51). Consequently, the UPR output was of a reduced synthesis of *Bip/Grp78* messenger RNA (mRNA) while there was an increased expression of ATF6-dependent *EDEM* mRNA, suggesting a negative role of CK2 on the ATF6 branch of the UPR (Fig. 2). More importantly from a therapeutic standpoint was the observation that the combined inactivation of CK2 (with a chemical inhibitor) and of Hsp90 (with 17-AAG) caused a synergistic cytotoxic effect on MM cells both in vitro and in vivo in mouse xenograft models. These data in MM reconcile with others’ data in different tumor cells demonstrating that CK2 maintains the ER stress response homeostasis [74]. Furthermore, CK2 seems critical for the ubiquitin proteasome clearance of proteins in MM and mantle cell lymphoma (MCL) [16]. When inhibited together with the proteasome, CK2 was found instrumental for MM and MCL cell survival as well as for the regulation of poly-ubiquitylated proteins. Bortezomib and CK2 inhibitors synergized in inducing MM cell death.

It has been demonstrated in some cancer cells that also CK1, together with CK2 and GSK3β, takes part in the regulation of Hsp70 and Hsp90, influencing their binding to the co-chaperones HOP (Hsp70-Hsp90 organizing protein) and the chaperone-binding ubiquitin ligase CHIP to determine the cellular protein folding/degradation balance [75]. The C-terminal phosphorylation of Hsp70 and Hsp90 in proliferating cancer cells enhances the assembly with HOP, increasing its protein folding activity. The non-phosphorylated chaperones preferentially bind to CHIP, mediating degradation of client proteins. It has been shown that melanoma, bladder, gastric, lung, breast, and pancreas cancer cells contain high levels of HOP, leading to high proliferation. Moreover, primary human breast tissue showed increased phosphorylated chaperones, compared to healthy samples. Even if a clear function of CK2 in UPR has been demonstrated in MM, given the role of CK1 on HOP and CHIP co-chaperones in other cancers, it is likely that also CK1α takes part in ER/stress, UPR in MM. Therefore, further research will have to clarify the exact role of CK1α in Hsp90-Hsp70 co-chaperone assembly and regulation in MM.

**Fig. 3:**
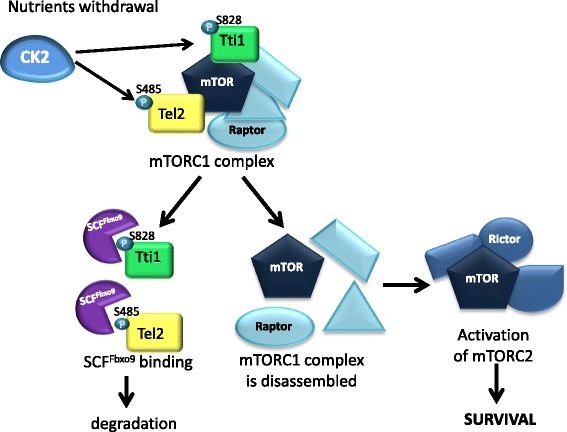
Schematic representation of the role of CK2 nutrients withdrawal. Upon nutrient withdrawal, CK2 phosphorylates Ser485 (S485) on Tel2 and Ser828 (S828) on Tt1, enabling the E3 ubiquitin ligase SCF^Fbxo9^ to target Tel2 and Tt1. The mTORC1 complex is therefore disassembled, and its inhibitory effect on mTORC2 is subsequently removed, allowing the cell to maintain the survival state

In the paper by Fernandez-Saiz et al. [76], a novel role for CK2 was discovered in relation to nutrients and growth factor withdrawal. The proteins telomere maintenance 2 (Tel2) and Tel2 interacting protein 1 (Tti1) were shown to be critical for the stability of PI3K-related kinase complex mammalian target of rapamycin complex 1 (mTORC1) by influencing assembly and maturation and were discovered to be targets of the E3-ubiquitin ligase SCF^Fbxo9^. In the absence of trophic signals, SCF^Fbxo9^ targets Tel2 and Tti1, thereby destabilizing mTORC1 complex. As a result, the feedback inhibition on the mTORC2 complex is relieved and the activity of the mTORC2 complex is sustained, allowing the cell to maintain the survival state. This SCF^Fbxo9^-dependent ubiquitination was found to be triggered by a CK2-executed priming phosphorylation of Ser485 on Tel2 and of Ser828 on Tti1 (Fig. 3). In roughly 30% of MM, SCF^Fbxo9^ was found overexpressed whereas Tel2 and Tti1 downexpressed. In these MM cases, AKT was overactive. Thus, CK2 might be a central regulator of AKT activity in a subset of MM.

### CK2 and CK1α role in bone marrow MM-stroma-delivered signals

**Fig. 4:**
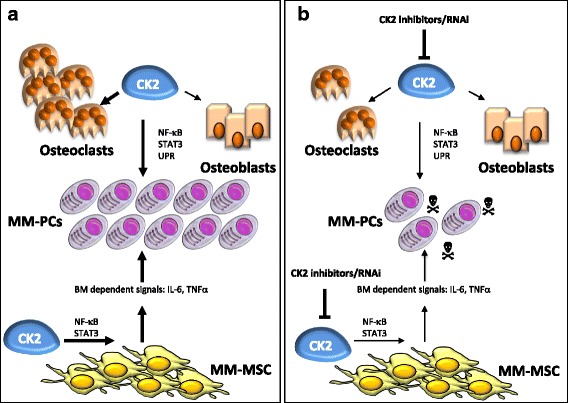
Schematic representation of the role of CK2 on MM-stroma-delivered signals. (**a**) CK2 sustains MM plasma cell survival through the activation of NF-ĸB, STAT3 and UPR. The activation of the kinase in BMSCs promotes the release of BM dependent signals (such as IL-6 or TNF-α) which in turn supports MM plasma cell survival. Moreover, CK2 sustains also osteoclast proliferation, pointing to a role of CK2 on MM-associated bone disease. (**b**) CK2 inactivation leads to MM osteoclast/BMSCs dependent plasma cell apoptosis

Our group has demonstrated that CK2 is contributory in supporting pivotal features of the BM microenvironment in MM [8]. The protective anti-apoptotic signals delivered by BMSCs are neutralized by CK2 inactivation. From a cellular and molecular standpoint, we observed that CK2 maintains a pro-survival signaling program from BMSCs to MM cells. Intriguingly, the inhibition of CK2 in BMSCs was accompanied by apoptosis of co-cultured MM cells, indicating the essentiality of the kinase in promoting growth signals from BMSCs towards MM cells. CK2 silencing in BMSCs caused a time-dependent inactivation of NF-κB and STAT3 in BMSCs and in MM cells. Indeed, the expression of IL-6 and TNF-α cytokines, mostly dependent on the activity of NF-κB and STAT3, was markedly reduced upon CK2 knockdown. An unforeseen result was the downregulation of the chemokine receptor CXCR4 in MM cells upon CK2 inhibition and consequently of the migratory potential of MM cells towards a concentration gradient of the respective ligand CXCL12 or SDF1α. The axis CXCL12/CXCR4 has been demonstrated to play an important role in MM cell homing in the protective BM *niche*, and inhibitors of CXCR4 are currently under scrutiny in clinical trials. Moreover, in this study, we provided evidence supporting a role for CK2 in bone homeostasis, which could be relevant in MM-associated bone disease. CK2 inhibition caused a relatively small cytotoxicity against human osteoblast cell line whereas it led to a dramatic reduction of human BM-derived osteoclast generation. Moreover, the osteoclast-dependent MM cell survival was reduced upon CK2 inhibition. The role of CK2 on MM-stroma-delivered signals is summarized in Fig. 4.

Regarding CK1α, this kinase provided a growth advantage in MM plasma cellular clone evolution by impinging on pivotal signalling cascades important for MM cell survival. In particular, by inhibiting PCs and bone BM survival signaling molecules (like β-catenin and AKT), CK1α silencing/inhibition determined MM cell apoptosis, even when co-cultured with BMSCs, overcoming stromal cell protection [17]. Moreover CK1α inhibition enhanced bortezomib and lenalidomide cytotoxicity on MM cells grown alone and/or on stromal cells, pointing to a function of CK1α on chemotherapy resistance. A recent study by Costa et al. [77] reported a role of CK1α in human dendritic cell (DC) maturation, modulating the mesenchymal stromal cell (MSC) inhibitory properties on DC evolution. CK1α silencing in MSC blunted the inhibitory effect of MSC on DC differentiation, increasing the expression of DC maturation markers (CD80, DC86, CD209) in a mechanism similar to that exerted by lenalidomide on DC of MM patients. This could suggest a potential role of CK1α in the modulation of MSC immunomodulatory properties. Further experiments would be necessary to prove this concept.

### CK1α and CK2 inhibition as a rational therapeutic approach in MM

Protein kinases are rational therapeutic targets since they are druggable with small molecule inhibitors. The proof-of-concept of the effectiveness of anti-kinase therapy was first shown in chronic myeloid leukemia, in which the inhibitor imatinib mesylate has successfully targeted the aberrant kinase activity of BCR-ABL1 [78]. Thereafter, a number of kinase inhibitors have entered the clinical arena. Recently, the B cell receptor signaling inhibitors idelalisib (which targets the PI3Kδ) and ibrutinib (which targets BTK) have shown an unprecedented clinical activity in B cell neoplasms [79, 80]. Nevertheless, in the anti-MM therapeutic armamentarium, kinase inhibitors are still lacking on the clinical ground.

The protein kinases CK1α and CK2 here discussed have demonstrated to play an important pro-survival role in MM. Thus, their inhibition could represent a rational approach in the therapy of this B cell malignancy. Recently, our group has shown that protein kinase CK2 sustains the growth of many blood cancers, including MM [71], MCL [16], DLBCL [81], and AML [82]. Moreover, the CK2 inhibitor CX-4945 is now in phase I clinical trial in solid tumors and relapsed/refractory MM. Clinical-grade, oral ATP-competitive small molecule CK2 inhibitors have been described and tested in preliminary clinical trials in solid tumors and also MM, even though no results of these trials have been published. These already available drugs or other compounds in development could be tested in combination therapies with conventional or novel agents. Indeed, CK2 inhibitors boost chemotherapy toxicity in cultured MM [71] and other hematologic [83–85] and solid tumor cells [63]. However, CK2 inhibition has proven to be a rational strategy also in combination with novel agents under study or already available in the therapy of MM. In particular, CK2 inhibition combined with the Hsp90 inhibitors geldanamycin or 17-AAG showed a remarkable in vitro and in vivo synergistic/cooperative cytotoxic activity in mouse models of MM [15]. Moreover, the association of the proteasome inhibitor bortezomib with K27 or CX-4945 showed a synergistic cytotoxic effect on MM and MCL cells [16].

Since CK2 inhibition is associated with the impairment of NF-κB and STAT3 activation and signaling, a rational use of CK2 inhibitors would be together with NF-κB and STAT3 inhibitors. In support of this approach, data from our laboratory have demonstrated that CK2 inhibitors and STAT3 or IKK inhibitors may cooperate in inducing cell killing of AML blasts [54]. If this holds true also in MM, which is a malignancy highly dependent on these two cascades, remains to be determined.

On the contrary, CK1α inhibition remains still an approach far from applicability in the clinical scenario, since no selective inhibitors targeting only this isoform of the CK1 family are available (D4476 is believed to be a dual CK1α/δ inhibitor). Nevertheless, the data here discussed support the idea that targeting CK1α might be beneficial at least in a subset of high-expressing MM patients. Moreover, it could always be possible to envision the dual CK1α/δ inhibition as an alternative approach, even if more data should be produced in this regard. Altogether, our in vitro studies indicate that CK1α inactivation may boost bortezomib and lenalidomide cytotoxicity. Further research should be pursued to confirm and extend these data.

## Conclusions

In conclusion, CK1a and CK2 are two Ser/Thr kinases whose role in controlling signaling pathways involved in proliferation, survival and stress resistance in MM has been robustly established. Moreover, the results highlight the role of CK1α and CK2 in sustaining BMSCs-delivered growth clues to MM cells suggesting that these kinases could represent targets to disrupt MM cell intrinsic as well as extrinsic survival mechanisms. Even though most of the data on the role of CK1α and CK2 in MM have been generated in preclinical experimental models, the strategy of inhibition of these two kinases appears to lay on a strong rationale. It could be anticipated that the inhibition of CK1α and/or CK2 could cooperate or synergize with conventional or novel anti-MM agents. CK1α and CK2 might therefore be taken into consideration for future therapeutic strategies in the treatment of this hard-to-defeat malignancy.

## Abbreviations

AML: Acute myeloid leukemia
ATR: Ataxia telangiectasia and Rad3-related protein
BM: Bone marrow
BMSCs: Bone marrow stromal cells
BTK: Bruton tyrosine kinase
CLL: Chronic lymphocytic leukemia
CML: Chronic myeloid leukemia
CX-4945: (3-Chlorophenylamino)benzo[c][2,6]naphthyridine-8-carboxylic acid)
D4476: (4-[4-(2,3-Dihydro-benzo[1,4]dioxin-6-yl)-5-pyridin-2-yl-1*H*-imidazol-2-yl]benzamide)
DLBCL: Diffuse large B cell lymphoma
EDEM: ER-degradation-enhancing-α-mannidose-like protein
ER: Endoplasmic reticulum
ERK: Extracellular signal-regulated kinase
FGF-3: Fibroblast growth factor 3
GSK3: Glycogen synthase kinase 3
HOP: Hsp70-Hsp90 organizing protein
IGF-I: Insulin-like growth factor-I
IL-6: Interleukin-6
IRF4: Interferon regulatory factor 4
MCL: Mantle cell lymphoma
MDS: Myelodisplatic syndromes
MM: Multiple myeloma
MSC: Mesenchymal stromal cells
mTORC1: Mammalian target of rapamycin complex 1
NF-κB: Nuclear factor kappa-light-chain-enhancer of activated B cells
PCs: Plasma cells
PI3K: Phosphoinositide 3-kinase
PTEN: Phosphatase and tensin homolog
RNAi: RNA interference
STAT3: Signal transducer and activator of transcription 3
Tel2: Telomere maintenance 2
TNF-α: Tumor necrosis factor-α
Tt1: Tel2 interacting protein 1
UPR: Unfolded protein response

## References

[1] Palumbo A, Anderson K (2011). Multiple myeloma. N Engl J Med.

[2] Podar K, Chauhan D, Anderson KC (2009). Bone marrow microenvironment and the identification of new targets for myeloma therapy. Leukemia.

[3] Manier S, Sacco A, Leleu X, Ghobrial IM, Roccaro AM (2012). Bone marrow microenvironment in multiple myeloma progression. J Biomed Biotechnol.

[4] Bahlis NP, NJ. (2012). Targeting of adhesion molecules as a therapeutic strategy in multiple myeloma. Curr Cancer Drug Targets.

[5] Hussein MA (2007). Multiple myeloma: most common end-organ damage and management. J Natl Compr Canc Netw.

[6] Kuehl WM, Bergsagel PL (2012). Molecular pathogenesis of multiple myeloma and its premalignant precursor. J Clin Invest.

[7] Corre J, Munshi N, Avet-Loiseau H (2015). Genetics of multiple myeloma: another heterogeneity level?. Blood.

[8] Manni S, Toscani D, Mandato E, Brancalion A, Quotti Tubi L, Macaccaro P (2014). Bone marrow stromal cell-fueled multiple myeloma growth and osteoclastogenesis are sustained by protein kinase CK2. Leukemia.

[9] Mitsiades CS, Mitsiades NS, McMullan CJ, Poulaki V, Kung AL, Davies FE (2006). Antimyeloma activity of heat shock protein-90 inhibition. Blood.

[10] Cea M, Cagnetta A, Adamia S, Acharya C, Tai Y-T, Fulciniti M (2016). Evidence for a role of the histone deacetylase SIRT6 in DNA damage response of multiple myeloma cells. Blood.

[11] Ocio EM, San Miguel JF (2010). The DAC system and associations with multiple myeloma. Investig New Drugs.

[12] Shaffer AL, Emre NCT, Lamy L, Ngo VN, Wright G, Xiao W (2008). IRF4 addiction in multiple myeloma. Nature.

[13] Cottini F, Hideshima T, Suzuki R, Tai Y-T, Bianchini G, Richardson PG (2015). Synthetic lethal approaches exploiting DNA damage in aggressive myeloma. Cancer Discov.

[14] Hurt EM, Thomas SB, Peng B, Farrar WL (2007). Integrated molecular profiling of SOD2 expression in multiple myeloma. Blood.

[15] Manni S, Brancalion A, Tubi LQ, Colpo A, Pavan L, Cabrelle A (2012). Protein kinase CK2 protects multiple myeloma cells from ER stress-induced apoptosis and from the cytotoxic effect of HSP90 inhibition through regulation of the unfolded protein response. Clin Cancer Res.

[16] Manni S, Brancalion A, Mandato E, Quotti Tubi L, Tubi LQ, Colpo A (2013). Protein kinase CK2 inhibition down modulates the NF-kappaB and STAT3 survival pathways, enhances the cellular proteotoxic stress and synergistically boosts the cytotoxic effect of bortezomib on multiple myeloma and mantle cell lymphoma cells. PLoS One.

[17] Manni S, Carrino M, Manzoni M, Gianesin K, Nunes SC, Costacurta M (2017). Inactivation of CK1alpha in multiple myeloma empowers drug cytotoxicity by affecting AKT and beta-catenin survival signaling pathways. Oncotarget.

[18] Knippschild U, Gocht A, Wolff S, Huber N, Lohler J, Stoter M (2005). The casein kinase 1 family: participation in multiple cellular processes in eukaryotes. Cell Signal.

[19] Knippschild U, Kruger M, Richter J, Xu P, Garcia-Reyes B, Peifer C (2014). The CK1 family: contribution to cellular stress response and its role in carcinogenesis. Front Oncol.

[20] Elyada E, Pribluda A, Goldstein RE, Morgenstern Y, Brachya G, Cojocaru G (2011). CKIalpha ablation highlights a critical role for p53 in invasiveness control. Nature.

[21] Pribluda A, Elyada E, Wiener Z, Hamza H, Goldstein RE, Biton M (2013). A senescence-inflammatory switch from cancer-inhibitory to cancer-promoting mechanism. Cancer Cell.

[22] Jaras M, Miller PG, Chu LP, Puram RV, Fink EC, Schneider RK (2014). Csnk1a1 inhibition has p53-dependent therapeutic efficacy in acute myeloid leukemia. J Exp Med.

[23] Cheong JK, Zhang F, Chua PJ, Bay BH, Thorburn A, Virshup DM (2015). Casein kinase 1alpha-dependent feedback loop controls autophagy in RAS-driven cancers. J Clin Invest.

[24] Bidere N, Ngo VN, Lee J, Collins C, Zheng L, Wan F (2009). Casein kinase 1alpha governs antigen-receptor-induced NF-kappaB activation and human lymphoma cell survival. Nature.

[25] Schittek B, Sinnberg T (2014). Biological functions of casein kinase 1 isoforms and putative roles in tumorigenesis. Mol Cancer.

[26] Sato Y, Yoshizato T, Shiraishi Y, Maekawa S, Okuno Y, Kamura T (2013). Integrated molecular analysis of clear-cell renal cell carcinoma. Nat Genet.

[27] Okerberg ES, Hainley A, Brown H, Aban A, Alemayehu S, Shih A (2016). Identification of a tumor specific, active-site mutation in casein kinase 1alpha by chemical proteomics. PLoS One.

[28] Dulak AM, Stojanov P, Peng S, Lawrence MS, Fox C, Stewart C (2013). Exome and whole-genome sequencing of esophageal adenocarcinoma identifies recurrent driver events and mutational complexity. Nat Genet.

[29] Kataoka K, Nagata Y, Kitanaka A, Shiraishi Y, Shimamura T, Yasunaga J-I (2015). Integrated molecular analysis of adult T cell leukemia/lymphoma. Nat Genet.

[30] Schneider RK, Adema V, Heckl D, Jaras M, Mallo M, Lord AM (2014). Role of casein kinase 1A1 in the biology and targeted therapy of del(5q) MDS. Cancer Cell.

[31] Hu Y, Song W, Cirstea D, Lu D, Munshi NC, Anderson KC (2015). CSNK1alpha1 mediates malignant plasma cell survival. Leukemia.

[32] Litchfield DW (2003). Protein kinase CK2: structure, regulation and role in cellular decisions of life and death. Biochem J.

[33] Gowda C, Song C, Kapadia M, Payne JL, Hu T, Ding Y (2017). Regulation of cellular proliferation in acute lymphoblastic leukemia by Casein Kinase II (CK2) and Ikaros. Adv Biol Regul..

[34] Piazza F, Manni S, Ruzzene M, Pinna LA, Gurrieri C, Semenzato G (2012). Protein kinase CK2 in hematologic malignancies: reliance on a pivotal cell survival regulator by oncogenic signaling pathways. Leukemia.

[35] Piazza F, Manni S, Semenzato G (2013). Novel players in multiple myeloma pathogenesis: role of protein kinases CK2 and GSK3. Leuk Res.

[36] Meggio F, Pinna LA (2003). One-thousand-and-one substrates of protein kinase CK2?. FASEB J.

[37] Di Maira G, Salvi M, Arrigoni G, Marin O, Sarno S, Brustolon F (2005). Protein kinase CK2 phosphorylates and upregulates Akt/PKB. Cell Death Differ.

[38] Guerra B (2006). Protein kinase CK2 subunits are positive regulators of AKT kinase. Int J Oncol.

[39] Channavajhala P, Seldin DC (2002). Functional interaction of protein kinase CK2 and c-Myc in lymphomagenesis. Oncogene.

[40] Romieu-Mourez R, Landesman-Bollag E, Seldin DC, Sonenshein GE (2002). Protein kinase CK2 promotes aberrant activation of nuclear factor-kappaB, transformed phenotype, and survival of breast cancer cells. Cancer Res.

[41] Landesman-Bollag E, Romieu-Mourez R, Song DH, Sonenshein GE, Cardiff RD, Seldin DC (2001). Protein kinase CK2 in mammary gland tumorigenesis. Oncogene.

[42] O-charoenrat P, Rusch V, Talbot SG, Sarkaria I, Viale A, Socci N (2004). Casein kinase II alpha subunit and C1-inhibitor are independent predictors of outcome in patients with squamous cell carcinoma of the lung. Clin Cancer Res.

[43] Laramas M, Pasquier D, Filhol O, Ringeisen F, Descotes J-L, Cochet C (2007). Nuclear localization of protein kinase CK2 catalytic subunit (CK2alpha) is associated with poor prognostic factors in human prostate cancer. Eur J Cancer.

[44] Stalter G, Siemer S, Becht E, Ziegler M, Remberger K, Issinger OG (1994). Asymmetric expression of protein kinase CK2 subunits in human kidney tumors. Biochem Biophys Res Commun.

[45] Zhang X, Yang X, Yang C, Li P, Yuan W, Deng X (2016). Targeting protein kinase CK2 suppresses bladder cancer cell survival via the glucose metabolic pathway. Oncotarget.

[46] Zhou B, Ritt DA, Morrison DK, Der CJ, Cox AD (2016). Protein kinase CK2alpha maintains extracellular signal-regulated kinase (ERK) activity in a CK2alpha kinase-independent manner to promote resistance to inhibitors of RAF and MEK but not ERK in BRAF mutant melanoma. J Biol Chem.

[47] Mandato E, Manni S, Zaffino F, Semenzato G, Piazza F (2016). Targeting CK2-driven non-oncogene addiction in B-cell tumors. Oncogene.

[48] Ruzzene M, Pinna LA (1804). Addiction to protein kinase CK2: a common denominator of diverse cancer cells?. Biochim Biophys Acta.

[49] Broseus J, Chen G, Hergalant S, Ramstein G, Mounier N, Gueant J-L (2016). Relapsed diffuse large B-cell lymphoma present different genomic profiles between early and late relapses. Oncotarget.

[50] Whitmarsh AJ (2011). Casein kinase 2 sends extracellular signal-regulated kinase nuclear. Mol Cell Biol.

[51] Ponce DP, Yefi R, Cabello P, Maturana JL, Niechi I, Silva E (2011). CK2 functionally interacts with AKT/PKB to promote the beta-catenin-dependent expression of survivin and enhance cell survival. Mol Cell Biochem.

[52] Park JH, Kim JJ, Bae Y-S (2013). Involvement of PI3K-AKT-mTOR pathway in protein kinase CKII inhibition-mediated senescence in human colon cancer cells. Biochem Biophys Res Commun.

[53] Ruzzene M, Bertacchini J, Toker A, Marmiroli S. Cross-talk between the CK2 and AKT signaling pathways in cancer. Adv Biol Regul. 2017;64:1–8. 10.1016/j.jbior.2017.03.002 28373060

[54] Quotti Tubi L, Canovas Nunes S, Brancalion A, Doriguzzi Breatta E, Manni S, Mandato E (2017). Protein kinase CK2 regulates AKT, NF-kappaB and STAT3 activation, stem cell viability and proliferation in acute myeloid leukemia. Leukemia.

[55] Silva A, Jotta PY, Silveira AB, Ribeiro D, Brandalise SR, Yunes JA (2010). Regulation of PTEN by CK2 and Notch1 in primary T-cell acute lymphoblastic leukemia: rationale for combined use of CK2- and gamma-secretase inhibitors. Haematologica.

[56] Romieu-Mourez R, Landesman-Bollag E, Seldin DC, Traish AM, Mercurio F, Sonenshein GE (2001). Roles of IKK kinases and protein kinase CK2 in activation of nuclear factor-kappaB in breast cancer. Cancer Res.

[57] Ponce DP, Maturana JL, Cabello P, Yefi R, Niechi I, Silva E (2011). Phosphorylation of AKT/PKB by CK2 is necessary for the AKT-dependent up-regulation of beta-catenin transcriptional activity. J Cell Physiol.

[58] Zhang S, Yang Y-L, Wang Y, You B, Dai Y, Chan G (2014). CK2alpha, over-expressed in human malignant pleural mesothelioma, regulates the Hedgehog signaling pathway in mesothelioma cells. J Exp Clin Cancer Res.

[59] Wang D, Westerheide SD, Hanson JL, Baldwin AS (2000). Tumor necrosis factor alpha-induced phosphorylation of RelA/p65 on Ser529 is controlled by casein kinase II. J Biol Chem.

[60] Seldin DC, Landesman-Bollag E, Farago M, Currier N, Lou D, Dominguez I (2005). CK2 as a positive regulator of Wnt signalling and tumourigenesis. Mol Cell Biochem.

[61] Gao Y, Wang HY (2006). Casein kinase 2 is activated and essential for Wnt/beta-catenin signaling. J Biol Chem.

[62] Zhang S, Wang Y, Mao J-H, Hsieh D, Kim I-J, Hu L-M (2012). Inhibition of CK2alpha down-regulates Hedgehog/Gli signaling leading to a reduction of a stem-like side population in human lung cancer cells. PLoS One.

[63] Siddiqui-Jain A, Bliesath J, Macalino D, Omori M, Huser N, Streiner N (2012). CK2 inhibitor CX-4945 suppresses DNA repair response triggered by DNA-targeted anticancer drugs and augments efficacy: mechanistic rationale for drug combination therapy. Mol Cancer Ther.

[64] Olsen BB, Wang S-Y, Svenstrup TH, BPC C, Guerra B (2012). Protein kinase CK2 localizes to sites of DNA double-strand break regulating the cellular response to DNA damage. BMC Mol Biol.

[65] Olsen BB, Svenstrup TH, Guerra B (2012). Downregulation of protein kinase CK2 induces autophagic cell death through modulation of the mTOR and MAPK signaling pathways in human glioblastoma cells. Int J Oncol.

[66] Buontempo F, Orsini E, Lonetti A, Cappellini A, Chiarini F, Evangelisti C (2016). Synergistic cytotoxic effects of bortezomib and CK2 inhibitor CX-4945 in acute lymphoblastic leukemia: turning off the prosurvival ER chaperone BIP/Grp78 and turning on the pro-apoptotic NF-kappaB. Oncotarget.

[67] Turowec JP, Duncan JS, Gloor GB, Litchfield DW (2011). Regulation of caspase pathways by protein kinase CK2: identification of proteins with overlapping CK2 and caspase consensus motifs. Mol Cell Biochem.

[68] Turowec JP, Vilk G, Gabriel M, Litchfield DW (2013). Characterizing the convergence of protein kinase CK2 and caspase-3 reveals isoform-specific phosphorylation of caspase-3 by CK2alpha': implications for pathological roles of CK2 in promoting cancer cell survival. Oncotarget.

[69] Parrish AB, Freel CD, Kornbluth S. Cellular mechanisms controlling caspase activation and function. Cold Spring Harb Perspect Biol. 2013;5:a008672. 10.1101/cshperspect.a008672 PMC3660825 23732469

[70] Kronke J, Fink EC, Hollenbach PW, MacBeth KJ, Hurst SN, Udeshi ND (2015). Lenalidomide induces ubiquitination and degradation of CK1alpha in del(5q) MDS. Nature.

[71] Piazza FA, Ruzzene M, Gurrieri C, Montini B, Bonanni L, Chioetto G (2006). Multiple myeloma cell survival relies on high activity of protein kinase CK2. Blood.

[72] Nishida MY, E. (2004). CK2 controls multiple protein kinases by phosphorylating a kinase-targeting molecular chaperone, Cdc37. Mol Cell Biol.

[73] Miyata Y (2009). Protein kinase CK2 in health and disease: CK2: the kinase controlling the Hsp90 chaperone machinery. Cell Mol Life Sci.

[74] Hessenauer A, Schneider CC, Gotz C, Montenarh M (2011). CK2 inhibition induces apoptosis via the ER stress response. Cell Signal.

[75] Muller P, Ruckova E, Halada P, Coates PJ, Hrstka R, Lane DP (2013). C-terminal phosphorylation of Hsp70 and Hsp90 regulates alternate binding to co-chaperones CHIP and HOP to determine cellular protein folding/degradation balances. Oncogene.

[76] Fernandez-Saiz V, Targosz BS, Lemeer S, Eichner R, Langer C, Bullinger L (2013). SCFFbxo9 and CK2 direct the cellular response to growth factor withdrawal via Tel2/Tti1 degradation and promote survival in multiple myeloma. Nat Cell Biol.

[77] Costa F, Vescovini R, Bolzoni M, Marchica V, Storti P, Toscani D, et al. Lenalidomide increases human dendritic cell maturation in multiple myeloma patients targeting monocyte differentiation and modulating mesenchymal stromal cell inhibitory properties: Oncotarget; 2017. doi:10.18632/oncotarget.18085. 10.18632/oncotarget.18085 PMC5581092 28881793

[78] An X, Tiwari AK, Sun Y, Ding P-R, Ashby CR, Chen Z-S (2010). BCR-ABL tyrosine kinase inhibitors in the treatment of Philadelphia chromosome positive chronic myeloid leukemia: a review. Leuk Res.

[79] Jerkeman M, Hallek M, Dreyling M, Thieblemont C, Kimby E, Staudt L. Targeting of B-cell receptor signalling in B-cell malignancies. J Intern Med. 2017. doi:10.1111/joim.12600. 10.1111/joim.12600 28295729

[80] Young RM, Shaffer AL, Phelan JD, Staudt LM (2015). B-cell receptor signaling in diffuse large B-cell lymphoma. Semin Hematol.

[81] Pizzi M, Piazza F, Agostinelli C, Fuligni F, Benvenuti P, Mandato E (2015). Protein kinase CK2 is widely expressed in follicular, Burkitt and diffuse large B-cell lymphomas and propels malignant B-cell growth. Oncotarget.

[82] Quotti Tubi L, Gurrieri C, Brancalion A, Bonaldi L, Bertorelle R, Manni S (2013). Inhibition of protein kinase CK2 with the clinical-grade small ATP-competitive compound CX-4945 or by RNA interference unveils its role in acute myeloid leukemia cell survival, p53-dependent apoptosis and daunorubicin-induced cytotoxicity. J Hematol Oncol.

[83] Prins RC, Burke RT, Tyner JW, Druker BJ, Loriaux MM, Spurgeon SE (2013). CX-4945, a selective inhibitor of casein kinase-2 (CK2), exhibits anti-tumor activity in hematologic malignancies including enhanced activity in chronic lymphocytic leukemia when combined with fludarabine and inhibitors of the B-cell receptor pathway. Leukemia.

[84] Martins LR, Perera Y, Lucio P, Silva MG, Perea SE, Barata JT (2014). Targeting chronic lymphocytic leukemia using CIGB-300, a clinical-stage CK2-specific cell-permeable peptide inhibitor. Oncotarget.

[85] Chon HJ, Bae KJ, Lee Y, Kim J (2015). The casein kinase 2 inhibitor, CX-4945, as an anti-cancer drug in treatment of human hematological malignancies. Front Pharmacol.

