# Correction to: Role of protein kinases CK1α and CK2 in multiple myeloma: regulation of pivotal survival and stress-managing pathways

**DOI:** 10.1186/s13045-018-0588-2

**Published:** 2018-04-05

**Authors:** Sabrina Manni, Marilena Carrino, Francesco Piazza

**Affiliations:** 1 Department of Medicine, Hematology Section, University of Padova, Padova, Italy; 2 Venetian Institute of Molecular Medicine, Padova, Italy

## Correction

The original article [1] contains an inadvertent error in the following sentence in the Abstract regarding the erroneous description of Ser/Thr kinases as ‘phylogenetically related’:

‘The phylogenetically related Ser/Thr kinases CSNK1A1 (CK1α) and CSNK2 (CK2) have recently gained a growing importance in hematologic malignancies arising both from precursors and from mature blood cells.’

The authors would like to note that the aforementioned Ser/Thr kinases are instead distantly related and that consequently, the sentence should read as the following instead:

‘The distantly related Ser/Thr kinases CSNK1A1 (CK1α) and CSNK2 (CK2) have recently gained a growing importance in hematologic malignancies arising both from precursors and from mature blood cells.’
